# Interventions for improving self-direction in people with dementia: a systematic review

**DOI:** 10.1186/s12877-021-02133-w

**Published:** 2021-03-20

**Authors:** Carola M. E. Döpp, Hermijntje Drenth, Paul-Jeroen Verkade, Anneke F. Francke, Iris van der Heide

**Affiliations:** 1 Nivel, Netherlands Institute of Health Services Research, P.O. Box 1568, 3500 Utrecht, BN Netherlands; 2 Stichting Geriant, Titanialaan 15A, 1702 Heerhugowaard, AZ Netherlands; 3 Amsterdam Public Health Research Institute, Amsterdam UMC, VU University Medical Center, Vrije Universiteit, P.O. Box 7057, 1007 Amsterdam, MB Netherlands

**Keywords:** Dementia, Alzheimer disease, Self-direction, Self-management

## Abstract

**Background:**

Dementia is a progressive disease that affects people’s everyday functioning, including the ability to express values, needs and wishes, which can be considered key elements of self-direction.

For the purpose of this review, self-direction refers to the organization and/or coordination of your own life, including professional and other care, with the objective of having what you perceive to be a good life. The aim of this systematic review was to assess and describe interventions that aim to improve self-direction of people with dementia.

**Methods:**

A systematic search was conducted in PubMed, Embase, CINAHL, PsycInfo and the Cochrane Library. Empirical studies up to April 2020 were included that used qualitative and/or quantitative methods and reported on interventions for people with dementia aimed at improving self-direction. Stepwise study selection and the assessment of methodological quality were conducted independently by two authors. Data on study and intervention characteristics, outcomes related to self-direction and well-being of people with dementia and factors influencing the feasibility were extracted systematically and described narratively.

**Results:**

Ten studies were identified describing a total of nine interventions. Interventions varied in terms of goals, content, target population and duration. Overall, interventions consisted of multiple components focusing on identifying “Who am I?” (beliefs, strengths, values, goals), identifying “What is important to me?” (meaningful activities and goal setting) and/or communicating about preferences with professionals and/or caregivers. The review provides indications that people with dementia may benefit from the interventions included. Overall, positive effects were found in studies on outcomes related to self-direction and wellbeing. However, outcomes measured using quantitative methods showed inconsistent effects between the studies.

**Conclusions:**

Although the methodological quality of all the studies included was ‘good’ or at least ‘fair’, the evidence base of interventions aiming to improve self-direction is still limited due to the low number of studies, the low number of participants and the frequent use of and their authors’ own non-standardized measures. Nevertheless, the review points towards positive effects on self-direction and well-being. Identifying individual beliefs, strengths, values, goals and meaningful activities can be essential components of these interventions, as well as communication about the desired care and support.

**Supplementary Information:**

The online version contains supplementary material available at 10.1186/s12877-021-02133-w.

## Background

Worldwide, around 50 million people have dementia and this is expected to increase to 152 million by 2050 [1]. Dementia causes deterioration in memory, thinking and the ability to perform everyday activities. It also reduces people’s ability to express their values, needs and wishes and to organize their lives accordingly [2, 3]. This ability is referred to as ‘self-direction’. In this paper, we refer to self-direction as organizing and/or coordinating your own life, including professional and other care, with the objective of having what you see as a good life. Self-direction revolves around the actual situation or expected situation in the future (adapted definition based on Verkooijen et al. [4]). Self-direction is closely related to various other terms such as self-determination, self-control, self-regulation and self-management [5, 6].

The self-direction of people with dementia is fundamental if professionals are to be able to adapt their care and support to the individual’s specific values, needs and wishes, and to be able to deliver effective care. Due to the progressive nature of dementia, the ability of people with dementia to express themselves and to practice self-direction diminishes over time [2, 3]. People with dementia therefore become increasingly dependent on their environment [7]. However, a study by Hamann et al. [8] shows that the choices that informal caregivers make do not always correspond to the wishes and needs of the person with dementia. This study revealed that people with dementia wanted to have more control over decisions than they were allowed by their caregivers [8].

As it can be difficult for people with dementia and their professional or informal caregivers to express or discuss values, wishes and needs by themselves, it can be helpful to use a tool or to follow an intervention programme that provides specific support. Various tools or programmes that assist self-direction in people with dementia and/or guide the discussion of values, wishes or needs with informal caregivers or healthcare professionals can be found in the literature [9]. However, no systematic review was available that summarized relevant tools and programmes. We will refer to tools, programmes and interventions hereinafter by the term ‘intervention’.

The aims of this review are to systematically identify and describe interventions that intend to improve self-direction in people with dementia and to report on the effects of these interventions and on the barriers and facilitators for implementing these interventions. The following research questions were formulated for this:

1. What interventions can be identified that aim to improve self-direction in people with dementia and that were evaluated in intervention studies? What are the main characteristics of these interventions?
2. What are the effects of these interventions on outcomes related to self-direction in people with dementia?
3. What are the effects of these interventions on the well-being of people with dementia?
4. What are the barriers and facilitators for implementing these interventions?

## Methods

### Design

The review protocol and the review report are based on the Preferred Reporting Items for Systematic Reviews and Meta-Analysis (PRISMA) statement [10, 11].

### Inclusion and exclusion criteria

#### Inclusion criteria

- The intervention study resulted in empirical qualitative and/or quantitative data.
- The research population consists of people with dementia. Additional actors, such as informal or formal caregivers may be included in the study population as well, but should not be the sole focus of the study.
- The intervention being studied aims to improve key elements of self-direction, in the sense of being involved in choices for organizing and/or coordinating activities aimed at a good life, including choices for organizing and/or coordinating your own professional or other care. Key elements of self-direction are considered to be the discussion of values, needs, wishes and preferences of people with dementia regarding their current and future life and care.
- The study describes results related to a) self-direction and/or b) well-being/quality of life and/or c) feasibility, facilitators for or barriers to the implementation process.

#### Exclusion criteria

- Studies of interventions solely involving instructions for specific medical treatments or medical decisions (e.g. regarding cardiopulmonary resuscitation preferences) were excluded.
- Group interventions that did not aim to actively encourage individual participants to engage in self-direction behaviour (e.g. peer support groups or information sessions that did not address elements of self-direction of individuals).
- Literature reviews (although their reference lists were studied to identify potential relevant underlying empirical studies).

No restrictions were applied on the publication date, in the selection process. Only studies published in English and Dutch were included.

### Search strategies and information sources

Searches were performed on 1 April 2020 in the databases PubMed, Embase, CINAHL, PsycInfo and Cochrane Library in collaboration with a medical librarian. Search terms included both controlled terms and free-text terms. In the Cochrane Library, only free-text terms were used and the search was conducted in all components of the library. The search string combined the terms ‘dementia’, ‘self-directing’, and ‘intervention’ with terms indicating an intervention study type. A full overview of the search strings used for each database can be found in Additional file 1. All identified references were entered into Endnote, after which duplicates were removed [12].

### Selection process

**Fig. 1:**
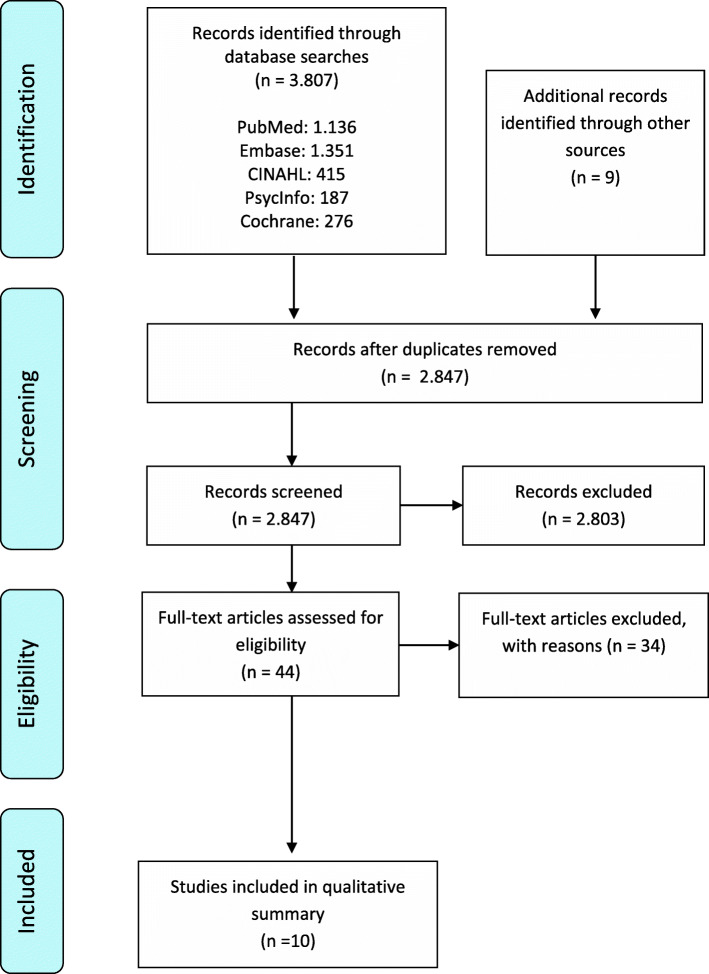
PRISMA Flow Chart

The references were entered in the software tool Covidence (https://www.covidence.org/) and then screened for relevance by two of the authors independently. Subsequently, the full texts of the remaining references were independently assessed by two authors. Disagreements between the reviewing authors were resolved by discussion. If necessary, a third reviewing author was consulted. The references were screened according to the predefined inclusion and exclusion criteria. A flow diagram of the selection process is shown in Fig. 1.

### Critical appraisal of the methodological quality

The critical appraisal instrument of Hawker et al. [13] was used to assess the quality of the included studies. This instrument was chosen as it can be used for studies with quantitative, qualitative and mixed-method designs. It consists of nine items: abstract and title, introduction and aims, methodology, sampling, data analysis, ethics and bias, results, transferability and implications. Each item was assessed for every study, with possible scores ranging from 1 (‘very poor’) to 4 (‘good’). The total score can range from 9 to 36 points. Scores of up to 18 are rated as ‘poor quality’, scores between 19 and 26 as ‘fair quality’ and scores of 27 or higher as ‘good quality’. The quality of each study was assessed independently by two researchers. If the overall quality scores of the two assessors differed by more than five points, the average was calculated (comparable to the approach used by Voss et al. [14]).

### Data extraction and analysis

Data was extracted about the study characteristics, intervention characteristics, study outcomes and the main conclusion as stated by the authors. For each of the studies, data was extracted by two of the authors. Results have been described narratively, as statistical pooling was impossible and the study designs and outcomes were rather heterogeneous and often qualitative in nature.

## Results

The various search strategies resulted in a total of 2847 unique references. Forty-four references remained after the first selection step (based on reading titles and abstracts). The full texts were then screened, after which another 34publications were excluded. The reasons for excluding these references are presented in Additional file 2. The two-step selection process ultimately resulted in 10 references eligible for inclusion, describing nine different interventions. The results of the selection process are presented in the PRISMA flow chart in Fig. 1. The two studies that evaluated the same intervention, the Early Diagnosis Dyadic Intervention (EDDI) [15, 16], differed in study design, outcome measures and sample sizes (see Table 1).

**Table 1:** Study Characteristics

AUTHOR (YEAR)	COUNTRY	AIM OF STUDY ACCORDING TO THE AUTHOR(S)	STUDY DESIGN	DATA COLLECTION METHOD	COMPARISON INTERVENTION(S)	STUDY POPULATION	OUTCOME MEASURES	NUMBER OF PARTICIPANTS AND THEIR CHARACTERISTICS	QUALITY (score)
**CONTROLLED STUDIES**									
**Hilgeman et al. (2014)** [17]	United States	To examine the impact of the Preserving Identity and Planning for Advance Care (PIPAC) intervention on coping strategies in early stages of dementia.	Two-group randomized controlled block design.	Qualitative and quantitative	Yes, a minimal support-based intervention focused on empathic listening and supportive reflection through two phone calls (10–30 min.). When no phone was available, brief face-to-face interactions were used.	People with early-stage dementia aged 55 and older and their family members/friends.	Depression and anxiety symptoms; quality of life; meaning in life; emotional support; health-related quality of life; perceptions of uncertainty in choosing future medical care; coping strategies for managing memory problems.	PIPAC group: 10 dyads. People with dementia: Mean age 80.8 (SD: 4.5); 7 female; mean years of education 13.9 (SD 3.4). Family contacts mean age 66.2 (SD: 11.3); 6 female; mean year of education 15.8 (SD 1.8). Minimal support group: 7 dyads and 1 person with dementia alone. People with dementia: Mean age 84.3 (SD: 8.0); 6 female; mean years of education 16.8 (SD 3.2). Family contacts mean age 68.6 (SD: 11.7); 5 female; mean year of education 16.3 (SD 1.8).	Good (35)
**Quinn et al. (2016)** [18]	United Kingdom	To explore the feasibility of a self-management intervention for people with early dementia. To make a preliminary assessment of whether the intervention enhanced participants’ sense of self efficacy. To explore the cost of setting up and delivering the intervention.	Two group randomized controlled trail.	Qualitative and quantitative	Yes, routine memory clinic services (nurse-led review and access to services such as psychiatry, psychology, occupational therapy, social services)	People with early-stage dementia and their family carers.	General self-efficacy measured with GSES; anxiety and depression measured with HADS; clinical outcomes measured with CORE-OM; health-related quality of life measured with the EQ-5D-3L; sense of capability-related well-being measured with ICECAP-O; intervention acceptability (attrition, adherence, satisfaction); costs of setting up and running the intervention.	24 people with dementia and their family carers (13 in intervention condition and 11 in control condition). Intervention group People with dementia: Mean age 75.2 (SD: 8.7); 3 female. Carers: Mean age 67.0 (SD: 15.0); 10 female. Control group People with dementia: Mean age 76.1 (SD: 8.5); 3 female. Carers: Mean age 66.2 (SD: 16.6); 9 female.	Good (33)
**Song et al. (2019)** [19]	United States	To adapt an efficacious Advanced Care Planning intervention, SPIRIT (sharing patient’s illness representations to increase trust), to be suitable for people with dementia and their surrogates. To assess whether people with dementia could meaningfully participate in the modified SPIRIT intervention and complete the outcome assessments.	Randomized controlled trial.	Qualitative	Yes, the modified version of SPIRIT for people with dementias was tested. A remote and in-person version were compared.	People with (mild or moderate) dementia and their surrogates.	Quantitative preparedness outcomes in the person with dementia and the surrogate: Dyad congruence in decision making, patient decisional conflict, and surrogate decision making confidence. Feasibility of SPIRIT - 1) tracking technical difficulties during SPIRIT remote sessions, 2) scoring PWD’s articulation of end-of-life care preferences, 3) rating their level of recollection of the SPIRIT session, and 4) tracking measures and items that PWDs found difficulty to understand. Acceptability and impact of SPIRIT - semi structured interview guide with each member of the dyad regarding their experience, perceived impact, facets that were experienced helpful or unhelpful, and suggestions for improvement.	23 people with dementia and their surrogates (11 in the remote group and 12 in the in-person group). Remote (intervention) group: People with dementia: Mean age 74.7 (SD: 7.9); 5 female. Surrogates: Mean age 69.7 (SD: 9.8); 7 female. Control (in-person) group: People with dementia: Mean age 73.7 (SD: 7.5); 7 female. Surrogates: Mean age 63.1 (SD: 11.8); 8 female.	Fair (23)
**Stockwell-Smith et al (2018)** [16]	Australia	To evaluate the effect of EDDI (Early Diagnosis Dyadic Intervention) a targeted community-based psychosocial intervention on self-efficacy outcomes for dementia dyads (person with dementia and their family carer) living with early-stage dementia.	Cluster randomized controlled design	Quantitative and qualitative.	Yes, control participants were provided with two information manuals.	Dyads of a person with early-stage dementia and their family carer.	Outcome measures was the self-rated self-efficacy (questionnaire). Data was collected at baseline, post-intervention (4 months’ postbaseline for the control group), and 4-month post-intervention. Post-intervention evaluation interviews were conducted with the dyad and the interventionist.	88 people with dementia and their surrogates (45 in the intervention group and 43 in the control group). Intervention (EDDI) group: People with dementia: mean age 77.2 (SD 7.7); 14 female. Carers: mean age 68.9 (SD 10.9); 37 female. Control group: People with dementia: mean age 78.5 (SD 6.4); 24 female. Carers: mean age 69.0 (SD 11.6); 34 female.	Good (28)
**UNCONTROLLED STUDIES**									
**Orsulic-Jeras et al. (2016)** [20]	United States	To describe the implementation of the Support, Health, Activities, Resources, and Education (SHARE) intervention, its unique core strategies and the potential impact SHARE can have on care planning for people with dementia and their families.	Before-and-after design.	Qualitative and quantitative	No	Dyads of person with early-stage dementia and their family carer.	Discussion of care needs; acknowledgement of the importance of a plan of care that takes into account the wishes of the person with dementia; acceptability of the program; feasibility of the program; decision-making and care-planning skills; benefits and drawbacks.	40 dyads: People with dementia (mean age 74.2 (SD: 8.7) 23 female) and caregivers (mean age 61.3 (SD: 15.8) 34 female).	Fair (27)
**Murphy & Oliver (2013)** [21]	United Kingdom	To explore whether Talking Mats could help people with dementia and family carers feel more involved in decisions making in their daily lives.	Crossover design.	Qualitative and quantitative	No	Dyads of person with dementia and their family carer.	Involvement Measure score; personal care; getting around; housework and activities.	18 dyads of person with dementia and family carer. Three participants were judged to have early-stage dementia, 13 moderate stage and 2 late-stage. Of the 18 family carers, 13 were female, with a mean age of 69 years (range 44–89).	Good (33)
**Poppe et al. (2013)** [22]	United Kingdom	To explore the acceptability of discussing advance care planning (ACP) with people with memory problems and mild dementia shortly after diagnosis.	One group post-test design.	Qualitative	No	People with mild dementia; family carers; staff from memory service and a community mental health team for older people.	Among people with dementia and carers: Issues around diagnosis, reason for ACP, evaluation of ACP discussion. Among staff: The ACP tool, the ACP discussion, barriers and facilitators for ACP, required skills and competencies.	12 people with dementia mean age 78.8 (SD: 6.2); 8 female; 6 living alone. 8 family carers, 4 female. 6 staff members from a memory clinic and a community mental health team.	Fair (27)
**FEASIBILITY / PROCESS EVALUATIONS**									
**Boyd (2007)** [9]	United Kingdom	To evaluate whether the Values History Form (VHF) can be used as a practical tool to empower people with dementia, should their condition deteriorate and they are no longer able to represent their views.	Process evaluation.	Qualitative	No	People with early-stage dementia and their potential carers as well as health care professionals (social workers, psychiatrists, GPs, CPNs, residential care staff, district nurses, nursing home staff and voluntary care managers).	Ability to discuss the future; Ability to discuss and record values; ideas about the VHF; Practical issues of engaging with the VHF.	Phase 1) 12 dyads of people with dementia (mean age 75.3 (SD: 7.1); 5 female) and family carers. Also 40 professionals working in dementia care.	Good (34)
**Martin et al. (2015)** [23]	United Kingdom	To evaluate the experiences with a self-management program and to perform an initial process evaluation.	Process evaluation.	Qualitative	No	People with early-stage dementia and course tutors.	Experiences; perceived benefits of the intervention; most/least useful program activities.	6 people with dementia living at home (mean age 68.9 (SD: 8.98); 3 female; mean dementia diagnosis of 3.5 years (SD: 3.5) and 2 course tutors.	Good (30)
**Whitlatch et al. (2006)** [15]	United States	To describe the Early Diagnosis Dyadic Intervention (EDDI) protocol, to report on the program’s acceptability, feasibility and usefulness and to discuss future applications of the protocol.	Feasibility study.	Quantitative	No	Dyads of person with early-stage dementia and primary responsibility family carer living in the same community with a close kin or kin-type relationship. Counselors were invited as well.	The measures of feasibility and acceptability included (a) the number of sessions attended by the caregiver and care receiver and the amount of time spent in the EDDI program; (b) care- givers’ and care receivers’ ratings of treatment satisfaction to assess the acceptability of the intervention and ratings by caregivers of their counselor’s effectiveness and enthusiasm; and (c) counselors’ ratings of their own enthusiasm; effectiveness; whether session goals were met.	2 counselors completed all nine sessions. 20 dyads completed all nine sessions.	Fair (26)

### Study characteristics

The characteristics of the 10 studies included are shown in Table 1. The studies were published between 2006 and 2019. Five studies were performed in the United Kingdom [9, 18, 21–23], four in the United States [15, 17, 19, 20] and one in Australia [16].

#### Study design, data collection methods and study quality

Three types of studies could be distinguished. In the first, randomized controlled studies were used that aimed to evaluate the effect of the intervention by comparison with a control intervention [16–19]. Three of these studies used both qualitative and quantitative methods to evaluate the intervention. The methodological quality of these three studies was scored as being ‘good’ (see Table 1).

The second type were uncontrolled studies that aimed to explore the potential impact and feasibility of an intervention [20–22]. These studies used qualitative methods and the methodological quality was scored as ‘fair’.

Thirdly, this review included studies that described the feasibility/process evaluation of an intervention [9, 15, 23]. Two of these studies were rated as ‘good’ in terms of methodological quality [9, 23]; both used qualitative methods to collect data. One was scored as having ‘fair’ methodological quality [15]. That study collected data using questionnaires and by collecting data about e.g. the number of sessions participants attended.

The measurement instruments varied between the studies. Self-developed measures were used in most studies. When pre-existing measurement instruments were used, they were most frequently instruments regarding well-being or quality of life (e.g. Quinn et al. [18]).

#### Setting and study population

All studies were performed in community settings and examined the effects of the intervention among people with early-onset or mild dementia. Three studies additionally evaluated the effect of the intervention among informal caregivers and healthcare professionals [9, 15, 22]; six studies additionally evaluated the effect of the intervention among informal caregiver [16–21]; and one study additionally evaluated the effect of the intervention among healthcare professionals [23]. Between 6 and 88 individuals with dementia were included in the study samples, with mean ages ranging from 68.9 (SD: 9.0) [23] to 80.8 (SD: 4.5) [17]. All studies included people with early-stage or mild dementia.

### Intervention characteristics

The 10 included studies covered a total of nine interventions. Characteristics of the interventions are shown in Table 2 and described in more detail in the following sections.

**Table 2:** Intervention Characteristics

AUTHOR (YEAR)	NAME OF THE INTERVENTION	AIM OF THE INTERVENTION	DESCRIPTION OF THE INTERVENTION	TARGET POPULATION OF THE INTERVENTION	TYPE OF INTERVENTION	INTENSITY / DURATION	TRAINER
**Boyd (2007)** [9]	Values History Form (VHF)	To empower people with dementia, enabling their voices to be heard should they no longer be able to articulate their wishes.	Values History is an approach during which core values and beliefs are identified that are important to the individual with a terminal illnesses as a basis for medical treatment should they lose capacity. The Values History Form (VHF) is filled out with the person with dementia and the family carer during five weekly interviews.	Community-dwelling people with early-stage dementia and their family carer	Interviews with individual dyads	Weekly interviews during five weeks	Researcher (psychiatric nurse)
**Hilgeman et al. (2014)** [17]	PIPAC	Maximize coping and enhance quality of life and well-being in the early stages of dementia.	Four sessions combining identity-maintaining activities (making a product such as a scrapbook or memory box through reminiscing activities) with an advance care planning (ACP) discussion (‘what it has meant to live well in the past’ and ‘what it will mean to live well in the future.’).	Community-dwelling people with early-stage dementia 55 years or older. Family contacts were where invited to, but this was not required.	In-home sessions with the person with dementia (and the informal caregiver)	Four in-home sessions during 4 to 6 weeks	Trained interventionist
**Martin et al. (2015)** [23]	Self-management intervention	To (a) help participants to communicate their feelings to family/friends; (b) feel more able to maintain an active lifestyle; (c) improve the experienced quality of life; (d) improve awareness of strategies for coping with changing memory and (e) improve knowledge in how to access relevant information.	The program addresses relationship with family, maintenance of an active lifestyle, psychological well-being, techniques to cope with memory changes and information about dementia. Sessions included relaxation, goal setting, action planning, goal feedback, problem solving, identifying personal strengths and maintaining a focus on engaging in pleasurable activities.	People with early-stage dementia	Group sessions (guided by a tutor’s manual) Handout Mid-week phone calls	Weekly group sessions of 2.5 h during six weeks.	co-delivery: Lay person and clinical professional tutors.
**Murphy & Oliver (2013)** [21]	Talking Mats	To support people with dementia and their carers in interacting with each other.	Talking Mats uses a simple system of picture symbols, placed on a textured mat, that allow people to indicate their feelings about various options within a topic by placing the relevant image below a visual scale. Sessions were held with dyad of person with dementia and their family carer to discuss issues around daily living by the use of Talking Mats.	People in all stages of dementia and their family carer	Tool to support interaction between person with dementia and their professional and informal carers.	Frequency and duration can be adapted ‘to the dyads needs. For the study, two in-home sessions were organized.	No trainer. For the study, a researcher facilitated discussion
**Orsulic-Jeras et al. (2016)** [20]	Support, Health, Activities, Resources, and Education intervention (SHARE)	To encourage care dyads to “work together” to discuss care value and preferences, address sensitive topics through education, strengthen communication skills, and ultimately develop a plan of care that can be adjusted as needed throughout the care path.	SHARE is a counseling-based care-planning intervention. It is based upon assessing and documenting care values and preferences for future care. The sessions are supported by a care values magnet board.	Dyads of people with early-stage dementia and their family carer	Individual dyad sessions.	Seven SHARE sessions were held over a period of four months.	Trained SHARE counselors
**Poppe et al. (2013)** [22]	Advanced Care Planning in Early Dementia Tool (ACP-ED)	To structure discussions between professional carers and people with dementia about advanced care planning (defined as the process of discussing an individual’s preferences for care they would like to receive at a time when they may no longer be able to make such decisions or their wishes known).	A written tool with eight questions for structuring ACP discussion. Questions concern need of information, wishes and needs for future support and care, needs and wishes regarding religion, culture, diet, involvement of family and friends in care provision, place of living when independent living is no longer possible, and additional needs.	People with early-stage dementia.	Written tool	Frequency and duration can be adapted by the dyads needs. For the study, two in-home sessions were organized.	No trainer
**Quinn et al. (2016)** [18]	Self-management program (SMART study)	To help people with dementia to practically manage their memory difficulties and to find ways of dealing with changes in their lifestyle.	Group sessions included providing information, enhancing self-efficacy, and encouraging vicarious learning. The facilitators provide information about dementia. To enhance group members’ sense of self-efficacy, the program encouraged group members to develop skills in problem-solving, goal-setting, and mindfulness-based relaxation. Additionally, the facilitators encouraged group members to share ideas, strategies, and achievements and so learn from each other. The first and last sessions were for both the person with dementia and the family carer; the other sessions were only for the person with dementia.	People with early-stage dementia and family carers.	Group sessions	Eight weekly 90-min group sessions.	Sessions were run by a staff nurse and a support worker.
**Song et al. (2019)** [19]	SPIRIT - Sharing Patient’s Illness Representations to Increase Trust	To promote preparation for end-of-life decisions in people with dementia and their surrogates.	SPIRIT was adapted to be suitable for people with dementia. The original SPIRIT is a structured psychoeducational intervention provided during a face-to-face interview. The intervention has six steps: 1) assessing illness representation, 2) identifying gaps and concerns, 3) creating conditions for conceptual change, 4) introducing replacement information, 5) summarizing, and 6) setting goals and planning. A Goals-of-Care tool is completed at the end of the session to indicate the patient’s preferences. The exact content of the modified version was not described.	Dyads of people with (mild to moderate) dementia and their surrogates.	Individual dyad sessions	One session (duration is not described in the original article).	The session was conducted by an trained social worker.
**Stockwell-Smith et al (2018)** [16]	The Early Diagnosis Dyadic Intervention (EDDI)	To assist people with dementia and their family carer to identify challenges they may experience during the dementia trajectory and to empower them to develop strategies and support structures to manage these challenges.	Modified version of the USA EDDI. Dyad members worked together and individually with a facilitator to 1) identify their current care values, 2) their future care needs and preferences, 3) discuss preferences for care providers, and 4) consider suitable care settings.	Dyads of people with early-stage dementia and their family carer.	Individual dyad sessions.	Seven weekly session. Each session lasting between 60 and 90 min.	Community practitioners recruited from two Queensland-based not-for-profit community service providers.
**Whitlatch et al. (2006)** [15]	The Early Diagnosis Dyadic Intervention (EDDI)	To help people with dementia and informal carers to express their preferences and concerns about the care situation and to strengthen the relationship bond.	During the individual sessions the tools used were a magnetic board, care values ladder worksheet, care preferences worksheet, barriers and solutions worksheet and care plan worksheet. The program’s objectives were (a) to increase the understanding of care preferences and values of each dyad member; (b) to discuss and practice effective communication techniques; (c) to discuss discrepancies in care preferences and expectations; (d) to increase the dyad’s knowledge about available services; and (e) to explore the emotional significance and relationship issues brought on by the illness for both care partners.	People with early-stage dementia and their informal caregivers	Individual dyad in-home sessions.	Nine structured individual 60–90 min in-home sessions. Five joint and four separate sessions delivered weekly or biweekly over an average period of 3 months (range 1.5 to 7.2 months).	Trained counselors.

#### Aims of the interventions

The most frequently mentioned aim of interventions was ‘improving self-direction’. This was the main aim of the interventions Value History Form (VHF by Boyd [9]), Preserving Identity and Planning for Advance Care (PIPAC by Hilgeman et al. [17]), the Support, Health, Activities, Resources and Education (SHARE by Orsulic-Jeras et al. [20]), the Sharing Patient’s Illness Representations to Increase Trust (SPIRIT by Song et al.*,* 2019) and the Early Diagnosis Dyadic Intervention (EDDI by Whitlatch et al. [15] and Stockwell-Smith et al. [16]). Additionally, improving self-direction was a secondary goal in the Self-management intervention [23], the SMART intervention [18], the Talking Mats [21] and the ACP-ED intervention [22].

The Self-management intervention [23] and the SMART intervention [18] were more broadly directed at living well with dementia, with improving self-direction as one of the elements.

The Talking Mats intervention [21] aimed to improve communication between people with dementia and their informal caregivers. The authors stress that the tool aims to include the perspective of the person with dementia more in both daily decisions and potential future decisions. The ACP-ED tool [22] focuses on advance care planning (ACP) and in that context on formulating wishes and preferences about care.

#### Content of the interventions

The interventions varied in terms of their content but all included multiple components. All the interventions had components for identifying what is important for the individual. These were activities such as identifying and recording the individual’s core values and beliefs [9], assessing and documenting care values, needs and/or preferences [15–17, 20, 22], exploring their own identity [17], setting goals [19] and identifying personal strengths [23] and meaningful activities [18, 23].

In addition, seven interventions facilitated discussion about the things that people with dementia identified as being important to them. These included discussions about the meaning of living well in the present and in the future [17], activities and goals [18–20, 23], care values and other values and preferences as identified by the person with dementia and/or the caregiver [20, 23], and the importance of certain predefined topics [15, 16, 21].

Three interventions specifically addressed communication [15–17, 23], for instance discussing challenges that people with dementia experience in communicating with professionals and how to address those challenges [23]. Another intervention included rehearsing communication about preferences with important loved ones [17]. The intervention described by Whitlatch et al. [15] and Stockwell-Smith et al. [16] included discussion and practice of effective techniques for communication between the person with dementia and the caregiver.

Three of the interventions [9, 20, 22] specifically addressed preferences of people with dementia in relation to the provision of current or future care. The other five encouraged self-direction in a broader sense with the goal of letting people to live as well as possible based on their own preferences.

#### Content of the control interventions

Four studies included a control intervention (see Table 1). These control interventions mainly consisted of minimal support: empathic listening and supportive reflection through two phone calls [17], routine memory clinic services [18] and information materials [16]. One study evaluated the feasibility of a remote (via video calling) versus an in-person version of an intervention to promote preparation for end-of-life decisions for patients and their caregivers [19].

#### Form of the interventions

The way the interventions were delivered varied. Five interventions concerned individual sessions for the person with dementia, while two interventions were based on group sessions. The two remaining interventions were tools intended for use by the person with dementia and an informal carer without support from a professional. The seven interventions using either individual or group sessions were all provided by a trained professional.

Sessions were often provided on a regular basis. Five interventions were provided weekly or fortnightly over periods varying between 4 and 12 weeks. One intervention provided seven sessions during a period of 4 months and one provided a single session. One of the two tools included symbols and pictures that support people with dementia in expressing their feelings [21]. The other tool was a guideline that helped to structure discussion between people with dementia and healthcare professionals [22].

### Study outcomes

Table 3 summarizes study outcomes related to self-direction and well-being in people with dementia and the feasibility, barriers or facilitators for the implementation process. These are discussed in more detail in the following sections.

**Table 3:** Study Outcomes

AUTHOR (YEAR)	OUTCOMES RELATED TO SELF-DIRECTION	OUTCOMES RELATED TO WELL-BEING	FEASIBILITY OUTCOMES	MAIN CONCLUSION ACCORDING TO THE AUTHOR(S)
**CONTROLLED STUDIES**				
**Hilgeman et al. (2014)** [17]	Intervention group individuals reported less overall conflict or discomfort with advanced care planning at post-treatment than the comparison group. Intervention group individuals reported feeling more supported in their decisions and feeling less distressed about incomplete information regarding decision-making. The uncertainty subscale and the values clarity subscale were not different between groups at post-treatment.	The intervention group reported less depressive symptoms, increased quality of life (BASQID), and better self-rated dependence in mobility at post-treatment compared to the comparison group. No differences were found regarding self-reported anxiety, meaning, social engagement, emotional anticipated support, self-care and quality of life measured by the QOL-AD, pain, and discomfort. Family contacts rated the intervention individuals as less depressed and as having higher quality of life (measured by the QOL-AD) than the comparison group. Family contact ratings of health-related indicators of well-being revealed differences on the EQ-5D domains of self-care, usual activities and anxiety/depression. Mobility, pain, discomfort and health status on a visual analog scale did not differ between groups.	Data on feasibility and acceptability indicated satisfactory acceptability, treatment implementation and feasibility among staff, people with dementia and family contacts.	The PIPAC intervention led to better outcomes than the comparison intervention across self- and proxy-reported measures for depression, QoL and health related indicators of well-being or illness burden.
**Quinn et al. (2016)** [18]	People with dementia stated that the intervention enabled them to express their point of view and that it enabled two-way conversations and discussion with informal carers.	Higher scores for QoL in the intervention group than in the control group; higher anxiety scores in intervention group than in the control group; lower depression scores in intervention group than in the control group; lower health-related QoL in the intervention group at three months, higher health-related QoL at six months in the intervention group.	People with dementia found the program enjoyable (75%) and helpful (92%) and would recommend it to others (75%). Most people wanted the program to last longer, and some participants would have preferred less heterogeneous participant groups. In terms of intervention acceptability, attrition was minimal, adherence was good, and satisfaction ratings were high.	This study has provided preliminary evidence that self-management may be beneficial for people with early-stage dementia. The program fostered independence and reciprocity, promoted social support, and provided information and help.
**Song et al. (2019)** [19]	Preparedness outcomes did not changes between baseline and post-intervention in both treatment groups. Dyad congruence was high, patient decisional conflict was low, and surrogate decision-making confidence was high. The most frequently stated benefit of SPIRIT was helping the person with dementia and the surrogate being on the same page.		*Feasibility of SPIRIT:* All 23 people with dementia were able to articulate their values and end of-life wishes. Nine people could not recall participating in SPIRIT at post-intervention. Several participant experienced some difficulty in choosing a response option in the Decisional Conflict Scale. Overall people with dementia stated that they felt comfortable during the session and that it felt good to express feelings and preferences. Surrogates appreciated the opportunity for themselves and PWDs to express their feelings and have open communication about dementia and end of life. Emotional difficulties were also reported. A few people with dementia found it hard to be confronted with death. For surrogates the most challenging part was the emotional difficulty visualizing the person with dementia’s end of life.	The adapted SPIRIT intervention enabled people with dementia in the study to engage in an advanced care planning (ACP) discussion and promoted authenticity of exchanges about experiences surrounding illness and values. Meaningful ACP conversations were possible even for those with moderate dementia and limited decision-making capacity.
**Stockwell-Smith et al (2018)** [16]	No significant between-group differences were found in Symptom Management or Support Service self-efficacy. *Service uptake:* Significant differences between groups in their use of education, training, and/or information services (at baseline and post intervention) and support group services (at 4 months follow-up). Most intervention dyads considered they were better informed about available support networks due to participation, “Gave us a broader appreciation of what’s involved and how we can use it”. And helped them prepare for changing needs: “The sessions helped us to decide to stay in our present house with modifications rather than move closer to family”.	Not addressed	Not addressed	The Self-Efficacy Questionnaire responses did not show a significant difference between the intervention and control groups over time. However, small but consistent improvements were found in the intervention group dyads’ uptake and awareness of community support and their use of education and information services and support group services. Improvements in self-efficacy were evident in the postintervention evaluation qualitative responses where dyads expressed greater confidence in identifying and accessing community support and expressed an increased awareness of their partner’s preferences regarding external care.
**UNCONTROLLED STUDIES**				
**Orsulic-Jeras et al. (2016)** [20]	People with dementia felt more in control of the care situation (88%), felt better prepared for what lies ahead (94%) and were more confident making care decisions (90%). Furthermore, 94% of the people with dementia felt that the SHARE program gave them an opportunity to express their thoughts and feelings.	Not addressed	80% of the caregivers and 65% of the people with dementia considered the number of sessions just right or would have liked more. Length of sessions was acceptable.	Planning in the early stages when people with dementia can voice their care values and preferences for future care is important.
**Murphy & Oliver (2013)** [21]	All participants felt significantly more involved in, and satisfied with discussions when they used Talking Mats. People mentioned that they were more aware of what they could still do and what they enjoyed doing, rather than just focusing on what they could no longer do.	Not addressed	Not addressed	People with dementia and family carers can use Talking Mats to feel more involved in making decisions about managing daily living. Talking Mats could result in increased well-being and positive adjustment to accepting care in people with dementia. The relationship between the person with dementia and the informal caregiver could be improved.
**Poppe et al. (2013)** [22]	People with dementia were relieved and felt less worried and more secure after discussing their preferences for the future. Some people found the discussion dispiriting and difficult without knowing what the future would bring.	Not addressed	Patients, staff, and carers agreed that all relevant issues were covered in the ACP-ED tool. Most carers and patients would recommend the tool. The relationship between patient and carer should be good and the diagnosis should be accepted before starting using the tool. Timing of the intervention is therefore dependent on the individual situation, but rather sooner than later.	The evaluation suggests that the ACP-ED tool can, with training, enable advanced care planning in people with mild dementia following diagnosis. This supports the implementation of advanced care planning in memory services and community mental health teams.
**FEASIBILITY / PROCESS EVALUATIONS**				
**Boyd (2007)** [9]	People in the early stages of dementia, particularly those with strong values, were able to articulate their values. Exploring other topics, such as reminiscing about family, friends and life situations, made their values apparent.	Not addressed	Half the participants did not have strong values and were happy to leave decisions for family members. Some of the participants felt threatened when discussing their diagnosis and future. The other half of the participants were happy to talk about and record their values. Some participants needed prompts from the interviewer or informal caregiver to articulate their values. The study showed that the vast majority of professionals would refer to the documents and find them useful. The majority would attempt to maintain past wishes and values, although not if it caused agitation or distress to the person. Many professionals stated that ‘time’ would be the main barrier for using the tool.	The study showed that the VHF can be an effective way of empowering people with dementia.
**Martin et al. (2015)** [23]	The task of setting goals was seen as something that focused thoughts on the future and planning to do something positive. There was a positive impact on self-esteem.	There is preliminary evidence that the intervention improves quality of life, psychological well-being, and coping with changes in memory.	The data supports the feasibility of the intervention: The goal setting activity was positively received by participants, participants were able to attend the sessions, complete activities and reported enjoyment and benefits from this. The program’s flexible nature, focus on strengths and the opportunity to spend time with other people living with dementia were particularly well received.	The results highlight the usefulness and acceptability of self-management for people with early-stage dementia and provide initial support for the program’s structure and content.
**Whitlatch et al. (2006)** [15]	Not addressed	Not addressed	Dyads on average attended 6.93/9 sessions and rated the sessions as highly acceptable and the enthusiasm and effectiveness of the counsellor as high. Counsellors were slightly less positive, but scores indicate that the counsellors believed that session goals and acceptability of the intervention were consistently achieved throughout the sessions. Some of the tools turned out to be challenging and should be simplified.	Participant and counsellor evaluations of the EDDI protocol indicated that the intervention was acceptable and satisfactory to the caregivers, care receivers and counsellors. Moreover, the intervention’s goals and objectives were achievable.

#### Outcomes related to self-direction in people with dementia

Nine out of the 10 included studies reported on outcomes related to self-direction, of which four included a control condition [16–19]. None of the controlled studies reported significant differences in measures related to self-direction between the intervention and the control group.

Both the controlled and uncontrolled studies uniformly reported positive evaluations for the intervention within the intervention group, for instance in terms of improved ability among people with dementia to express and discuss feelings, thoughts and preferences [18–22]. Furthermore, people with dementia experienced less discomfort with advance care planning, felt more supported in their decisions and more prepared for changing needs [16, 17]. People who used the Talking Mats mentioned that they were more aware of what they could still do and what they enjoyed doing, rather than just focusing on what they could no longer do [21]. Other positive results of the interventions reported were feeling less worried and more secure [22], feeling more in control of the care situation and feeling better prepared for what lies ahead [20].

Less positive findings were that some participants found the discussion about advance care planning dispiriting and difficult without knowing what the future would bring [22].

Two of the feasibility studies reported on self-direction [9, 23]. Boyd [9] reported that people in the early stages of dementia, particularly those with strong values, were able to articulate their values. Exploring other topics, such as reminiscing about family, friends and life situations, made their values apparent. Martin et al. [23] reported that people gave a positive evaluation of the task of setting goals and thereby planning for the future and reported a positive impact of this task on their self-esteem.

#### Outcomes related to well-being in people with dementia

Three studies reported on outcomes related to the well-being of people with dementia, including two controlled studies [17, 18] and one feasibility study [23]. The controlled studies showed that people with dementia in the intervention group reported fewer depressive symptoms, increased (health-related) quality of life and better self-rated dependence in mobility compared to the control group after receiving the intervention [17, 18].

One of these controlled studies found no effect of the intervention on anxiety, social engagement, emotional and anticipated support in the intervention group compared to the control group [17], whereas the other study found higher anxiety scores 3 months after the intervention in the intervention group compared to the control group [18].

The feasibility study by Martin et al. reported that people with dementia experience better psychosocial well-being, improvement in coping with memory loss and improved quality of life [23].

#### Outcomes related to the feasibility of interventions

Eight studies reported on the feasibility of the interventions [9, 15, 17–20, 22, 23]. Consent rates ranged from 66% [19] to 82% [20] for most of these studies. The study by Quinn et al. had a consent rate of 17% [18] and the study by Hilgeman et al*,* had one of 28% [17]. The most common reasons for drop-out that were reported by some of these studies included health problems, family stress and lack of time [17, 20, 23].

The acceptability of interventions was rated as high by the participants of the studies by Martin et al.*,* Hilgeman et al. and Orsulic-Jeras et al. [17, 20, 23]. Martin et al. [23] reported that participants were especially satisfied with the flexible nature of the programme, the focus on strengths and the opportunity to spend time with other people living with dementia. Orsulic-Jeras et al., on the other hand, reported several drawbacks with respect to the SHARE intervention that hamper the acceptability of this intervention: the length (too long or too short), stress (related to topics discussed), irrelevant programme and dissatisfaction [20]. Although their study showed that significantly fewer drawbacks than benefits were reported for the intervention, it also implied that not all participants are ready to discuss certain topics early in the care pathway [20].

It becomes clear from several studies that it depends very much on the individual whether they feel comfortable talking about their wishes, needs and values. Both Boyd et al. [9] and Song et al [19] found for instance that most of their study participants were willing to talk about their values; however, other participants felt threatened or uncomfortable talking about values in relation to their diagnosis. In addition, participants found it difficult in some cases to be confronted with death [19]. Orsulic-Jeras et al. [20] also reported that not all participants felt the need to express thoughts and feelings when implementing the SHARE intervention.

The feasibility of the interventions is (from the professionals’ point of view) determined by the availability of time to implement the intervention. Boyd [9] reported for instance that professionals stated that a lack of time would be the main barrier to using the tool. From the point of view of people with dementia and their caregivers, the feasibility is strongly determined by the extent to which people with dementia are able to use the tool. Whitlatch et al. [15] for instance found that the tools they used during the sessions were sometimes too challenging for people with dementia.

## Discussion

This review explored the evidence base of interventions aimed at improving self-direction in people with dementia. Ten studies were identified, studying nine different interventions. The interventions varied in terms of their specific goals, content, target population and duration. Overall, the interventions consisted of one or more of the following three components 1) identifying “Who am I?” (beliefs, strengths, values, goals), 2) identifying “What is important to me?” (meaningful activities and goal setting) and 3) discussing what is important to them with professionals and/or caregivers.

Overall, this review suggests positive effects in qualitative terms of the studied interventions on self-direction and on the well-being of people with dementia. For example, participants were in general better able to express feelings, thoughts and preferences, had increased self-esteem and self-confidence, and felt more involved. In addition, studies revealed valuable lessons for the implementation of interventions to improve self-direction in people with dementia.

However, this review primarily shows that the evidence base of interventions aimed at self-direction of people with dementia is still very limited. The interventions that were included in this review often relate to advance care planning in people with dementia. Although attention is increasingly being paid to advance care planning interventions for people with dementia, this field is also characterized by limited evidence about the effectiveness of interventions (see for instance [24–26]). With respect to intervention studies on advance care planning in general and with respect to studies on self-direction specifically, there seems to be a lack of uniformly used measures for assessing outcomes [26].

The current review shows that measures of self-direction were often self-developed and not validated among the target population. These measures might therefore have not been sensitive enough to detect changes. It is therefore recommended that more research should be carried out into how self-direction in people with dementia can be adequately and uniformly measured, using insights from validated instruments such as the GAS and the COPM [27, 28].

Although the studies were good or fair in their methodological quality, the number of participants in all the studies was low. This might be related to the qualitative nature of most of the studies. Another reason for the small sample sizes might be the commonly experienced challenge of recruiting people with dementia for research [29]. As the number of people with dementia is still increasing exponentially, it is essential to find effective ways to increase participation in research.

Furthermore, current review shows that interventions so far focus on people with early-onset dementia. On the one hand, it is important to start conversations about values and wishes in an early stage of the disease, when the person with dementia is still able to articulate their values and wishes and to make decisions. On the other hand, we found that several studies as included in this review, report that both people with dementia as well as their caregivers often found it difficult and emotional to talk about future needs and to be confronted with death in the early stage of the disease. An important next step would therefore be to further explore possibilities and interventions to encourage self-direction later on in the disease pathway. There have been recent developments in this respect [24].

Another recommendation based on current review is to further study how interventions that are currently available meet the needs of people with dementia who have an immigration background. The studies that were included in the current review did not report on including respondents with varying cultural backgrounds. This means it remains unclear how suitable the interventions as described in this review are for people with dementia from migrant backgrounds. Given that many countries now have multi-cultural societies, exploring how the interventions can be provided in a culturally sensitive way is an important next step to take within the research field.

Despite the limited evidence base, the review does provide information about aspects that should be taken into account when developing and implementing an intervention to improve self-direction in people with dementia. For example, it is important to create a comfortable atmosphere for people to talk about their wishes, needs and values, to take into account the amount of time it costs for professionals and whether that is feasible and to make sure there is a fit between the interventions and the capacity, needs and interests of the participants. To improve the feasibility of interventions, we recommend that they should be developed or adapted in close collaboration with people with dementia. These interventions, including the materials used, should be tested prior to or as a separate phase in the development process. A participatory action research design might be suitable for this.

In addition, we found that all the interventions were multi-component and can therefore considered to be ‘complex’ interventions. Complex interventions should preferably be developed and evaluated using a systematic, step-by-step approach. An example of such an approach is the MRC framework for complex interventions [30, 31].

### Strengths and limitations

The current systematic review was novel in that it focused on interventions that aimed to improve self-direction among people with dementia, using a systematic approach. Self-direction of people with dementia is fundamental for professionals if they are to be able to adapt their care and support to the individual’s specific values, needs and wishes, and to be able to deliver effective care.However, a limitation of this review was that interventions were only included when they were part of empirical intervention research. There may be various interventions available that are used in clinical practice that have not been evaluated in research and which therefore cannot be found in scientific literature databases.

In addition, the concept of ‘self-direction’ (and related concepts such as self-determination, self-control, self-regulation, self-management and advance care planning) is a complex concept to define. It was a challenge to formulate specific definitions that did not overlap with other concepts. In addition, ‘self-direction’ as a term was not always explicitly used in the studies that were included. Instead, terms like self-determination, self-control, self-regulation, self-management or advance care planning were sometimes used. We therefore selected studies based on a broad inclusion criterion, namely that “the intervention being studied aims to improve key elements of self-direction, in the sense of being involved in choices for organizing and/or coordinating activities aimed at a good life, including choices for organizing and/or coordinating your own professional or other care.”

## Conclusion and recommendations

Although all included studies were ‘good’ or at least ‘fair’ in terms of methodological quality, the evidence base of interventions that aim to improve self-direction is still limited due to the low number of studies, the low number of participants and the frequent use of self-developed and non-standardized measures. Nevertheless, the review suggests positive effects on self-direction and well-being. Identifying individual beliefs, strengths, values, goals and meaningful activities, as well as noting that communication about the desired care and support can be essential components of these interventions.

To create a broader evidence base, we recommend that addition studies should be conducted into the effectiveness and feasibility of interventions to increase self-direction in people with dementia. Interventions should be developed or adapted in close collaboration with professionals, caregivers and people with dementia to make sure the interventions fit their needs. More attention should be paid to developing and/or using standardized and sensitive outcome measures for self-direction.

## Supplementary Information

**Additional file 1.** Search queries for each database.

**Additional file 2.** Full Text Exclusions with Reasons.

## Data Availability

The datasets used and/or analyzed during the current study are available from the corresponding author on reasonable request.

## Abbreviations

PRISMA: Preferred Reporting Items for Systematic Reviews and Meta-Analysis
VHF: Value History Form
SHARE intervention: Support, Health, Activities, Resources, and Education intervention
EDDI Intervention: Early Diagnosis Dyadic intervention
ACP-ED: Advanced Care Planning in Early Dementia
QoL: Quality of life
