# Supplementary material for: Interventions for improving self-direction in people with dementia: a systematic review
Source: BMC Geriatr. 2021 Mar 20;21:195. doi: 10.1186/s12877-021-02133-w (PMC7981798; doi:10.1186/s12877-021-02133-w)
Supplement: Supplementary file 1 — Additional file 1. Search queries for each database. [file 12877_2021_2133_MOESM1_ESM.docx]

# Additional file 1 – Search queries for each database

**PubMed History April 1st, 2020**

| Search | Query |
| --- | --- |
| #5 | #1 AND #2 AND #3 AND #4 |
| #4 | "Feasibility studies" [Mesh] OR "Evaluation Studies" [Publication Type] OR "Evaluation Studies as Topic"[Mesh] OR "Clinical Studies as Topic"[Mesh] OR randomized controlled trial[pt] OR controlled clinical trial[pt] OR randomized controlled trials[mh] OR clinical trial[pt] OR clinical trials[mh] OR trial*[tiab] OR feasib*[ tiab] OR evaluat*[tiab] OR effect*[tiab] |
| #3 | intervention*[tiab] OR program*[tiab] OR tool*[tiab] OR support*[tiab] |
| #2 | "Self-Management"[Mesh] OR "Patient Participation"[Mesh] OR "Decision Making"[Mesh] OR "Personal Autonomy"[Mesh] OR "Power (Psychology)"[Mesh] OR "Advance Care Planning"[Mesh] OR "Decision Support Techniques"[Mesh] OR self manage*[tiab] OR selfmanag*[tiab] OR self-direct*[tiab] OR selfdirect*[tiab] OR decision making*[tiab] OR decisionmaking*[tiab] OR sdm[tiab] OR advance care planning[tiab] OR autonomy[tiab] OR autonomous[tiab] OR mastery[tiab] OR desire*[tiab] OR personhood[tiab] OR selfhood[tiab] OR empower*[tiab] OR patient participation[tiab] OR patient engagement[tiab] OR patient involvement[tiab] |
| #1 | "Dementia"[Mesh] OR alzheimer*[tiab] OR dementi*[tiab] |

**Embase History April 1st, 2020**

| Search | Query |
| --- | --- |
| #6 | #5 NOT ('conference abstract'/it OR 'conference review'/it) |
| #5 | #1 AND #2 AND #3 AND #4 |
| #4 | 'intervention study'/exp OR 'evaluation study'/exp OR 'clinical study'/exp OR 'feasibility study'/exp OR feasibil*:ab,ti,kw OR evaluat*:ab,ti,kw OR trial*:ab,ti,kw OR effect*:ab,ti,kw |
| #3 | intervention*:ab,ti,kw OR program*:ab,ti,kw OR tool*:ab,ti,kw OR support*:ab,ti,kw |
| #2 | 'self directedness'/exp OR 'self care'/exp OR 'patient participation'/exp OR 'decision making'/exp OR 'empowerment'/exp OR 'advance care planning'/exp OR 'decision support system'/exp OR ‘self-manage*’:ab,ti,kw OR selfmanag*:ab,ti,kw OR ‘self-direct*’:ab,ti,kw OR selfdirect*:ab,ti,kw OR (decision* NEAR/3 making):ab,ti,kw OR decisionmaking*:ab,ti,kw OR sdm:ab,ti,kw OR ‘advance care planning’:ab,ti,kw OR autonomy:ab,ti,kw OR autonomous:ab,ti,kw OR mastery:ab,ti,kw OR desire*:ab,ti,kw OR personhood:ab,ti,kw OR selfhood:ab,ti,kw OR empower*:ab,ti,kw OR (patient NEAR/3 (participation OR engage* OR involv*)):ab,ti,kw |
| #1 | 'dementia'/exp OR 'Alzheimer disease'/exp OR alzheimer*:ab,ti,kw OR dementi*:ab,ti,kw |

**PsycInfo History April 1st, 2020**

| Search | Query |
| --- | --- |
| #5 | #1 AND #2 AND #3 AND #4 |
| #4 | DE ("Evaluation" OR "Program Evaluation" OR "Clinical Trials") OR (ZC "clinical trial") OR TI (evaluation OR feasibil* OR effect* OR trial*) OR AB (evaluation OR feasibil* OR effect* OR trial*) |
| #3 | DE ("Intervention") OR TI (intervention* OR program* OR tool* OR support*) OR AB (intervention* OR program* OR tool* OR support*) |
| #2 | DE ("Self-Management" OR "Client Participation" OR "Decision Making" OR "Autonomy" OR "Empowerment" OR "Decision Support Systems") OR TI ("self manage*" OR selfmanag* OR "self-direct*" OR selfdirect* OR decision making* OR decisionmaking* OR sdm OR advance care planning OR autonomy OR autonomous OR mastery OR desire* OR personhood OR selfhood OR empower* OR patient participation OR patient engagement OR patient involvement) OR AB ("self manage*" OR selfmanag* OR "self-direct*" OR selfdirect* OR selfcare OR "self care" OR decision making* OR decisionmaking* OR sdm OR advance care planning OR autonomy OR autonomous OR mastery OR desire* OR personhood OR selfhood OR empower* OR patient participation OR patient engagement OR patient involvement) |
| #1 | DE ("Dementia" OR "Alzheimer's Disease") OR TI (alzheimer* OR dementi*) OR AB (alzheimer* OR dementi*) |

**CINAHL History April 1st, 2020**

| Search | Query |
| --- | --- |
| #5 | #1 AND #2 AND #3 AND #4 |
| #4 | MH ("Clinical Trials+" OR "Evaluation Research+" OR "Evaluation" OR "Program Evaluation" OR "Usability Study")  OR ZT ("clinical trial" OR "randomized controlled trial") OR TI (evaluat* OR effect* OR trial* OR feasibil*) OR AB (evaluat* OR effect* OR trial* OR feasibil*) |
| #3 | TI (intervention* OR program* OR tool* OR support*) OR AB (intervention* OR program* OR tool* OR support* OR trial*) |
| #2 | MH ("Self Care" OR "Consumer Participation" OR "Decision Making+" OR "Autonomy" OR "Patient Autonomy" OR "Empowerment") OR TI (“self manage*” OR selfmanag* OR “self-direct*” OR selfdirect* OR decision making* OR decisionmaking* OR sdm OR advance care planning OR autonomy OR autonomous OR mastery OR desire* OR personhood OR selfhood OR empower* OR patient participation OR patient engagement OR patient involvement) OR AB (“self manage*” OR selfmanag* OR “self-direct*” OR selfdirect* OR decision making* OR decisionmaking* OR sdm OR advance care planning OR autonomy OR autonomous OR mastery OR desire* OR personhood OR selfhood OR empower* OR patient participation OR patient engagement OR patient involvement) |
| #1 | MH ("Dementia" OR "Alzheimer's Disease") OR TI (alzheimer* OR dementi*) OR AB (alzheimer* OR dementi*) |

**Cochrane Library History April 1st, 2020**

| Search | Query |
| --- | --- |
| #5 | #1 AND #2 AND #3 AND #4 |
| #4 | trial* OR feasibil* OR evaluat* OR effect* |
| #3 | intervention* OR program* OR tool* OR support* |
| #2 | “self manage*” OR selfmanag* OR “self-direct*” OR selfdirect* OR “decision making*” OR decisionmaking* OR “decision support technique*” OR sdm OR “advance care planning” OR autonomy OR autonomous OR mastery OR desire* OR personhood OR selfhood OR empower* OR (patient NEAR/3 (participation OR engagement OR involvement)) |
| #1 | Dementia* OR alzheimer* |
