# Supplementary material for: Interventions for improving self-direction in people with dementia: a systematic review
Source: BMC Geriatr. 2021 Mar 20;21:195. doi: 10.1186/s12877-021-02133-w (PMC7981798; doi:10.1186/s12877-021-02133-w)
Supplement: Supplementary file 2 — Additional file 2. Full Text Exclusions with Reasons. [file 12877_2021_2133_MOESM2_ESM.docx]

# Additional file 2 – Full Text Exclusions with Reasons

| Publication | Reason for exclusion |
| --- | --- |
| [Agar *et al.* (2017)](#_ENREF_2) | Target population (intervention) did not meet criteria |
| [Ballard (2010)](#_ENREF_7) | Type of publication did not meet criteria |
| [Barclay, Cherry, and Mittman (2006)](#_ENREF_8) | Target population (intervention) did not meet criteria |
| [Blokland, Asch, Doornaar, and Pot (2015)](#_ENREF_9) | Type of publication did not meet criteria |
| [Brazil *et al.* (2017)](#_ENREF_12) | Type of publication did not meet criteria |
| [Brazil, Carter, McLaughlin, Kernohan, Hudson, Clarke, Froggatt, *et al.* (2016)](#_ENREF_13) | Type of publication did not meet criteria |
| [Brazil, Carter, McLaughlin, Kernohan, Hudson, Clarke, Passmore, *et al.* (2016)](#_ENREF_14) | Type of publication did not meet criteria |
| [Caplan, Meller, Squires, Chan, and Willett (2006)](#_ENREF_16) | Outcome measures did not meet criteria |
| [Chang (2016)](#_ENREF_17) | Intervention did not meet criteria |
| [Chung (2001)](#_ENREF_18) | Intervention did not meet criteria |
| [Clare, Rowlands, and Quin (2008)](#_ENREF_19) | Intervention did not meet criteria |
| [Deep, Hunter, Murphy, and Volandes (2010)](#_ENREF_22) | Target population did not meet criteria |
| [Detering, Hancock, Reade, and Silvester (2010)](#_ENREF_24) | Target population did not meet criteria |
| [Ding *et al.* (2010)](#_ENREF_25) | No empirical data |
| [Einterz, Gilliam, Chang Lin, McBride, and Hanson (2014)](#_ENREF_29) | Target population did not meet criteria |
| [Famakinwa (2010)](#_ENREF_30) | Study design did not meet criteria |
| [Hanson, Song, *et al.* (2016)](#_ENREF_34) | Target population did not meet criteria |
| [Hanson, Zimmerman, *et al.* (2016)](#_ENREF_35) | Target population does not meet criteria |
| Jennings *et al.* (2019) | Outcome measures did not meet criteria |
| [Kerkhof, Graff, Bergsma, Vocht, and Droes (2016)](#_ENREF_41) | Type of publication did not meet criteria |
| [Lawani *et al.* (2017)](#_ENREF_42) | Type of publication did not meet criteria |
| Mitchell SL *et al.* (2018) | Target population did not meet criteria |
| [Molloy *et al.* (2000)](#_ENREF_48) | Intervention did not meet criteria |
| [Morrison *et al.* (2005)](#_ENREF_49) | Target population did not meet criteria |
| Mueller *et al.* (2018) | Intervention did not meet criteria |
| [Nomura *et al.* (2009)](#_ENREF_52) | Intervention did not meet criteria |
| [Romero and Wenz (2001)](#_ENREF_59) | Intervention did not meet criteria |
| [Sampson *et al.* (2011)](#_ENREF_60) | Intervention did not meet criteria |
| [Sprange *et al.* (2015)](#_ENREF_63) | Intervention did not meet criteria |
| Song *et al* (2018) | Type of publication did not meet criteria |
| [Villars, Dupuy, Perrin, Vellas, and Nourhashemi (2015)](#_ENREF_66) | Intervention did not meet criteria |
| [Volandes *et al.* (2011)](#_ENREF_67) | Outcome measures did not meet criteria |
| [Volandes, Mitchell, Gillick, Chang, and Paasche-Orlow (2009)](#_ENREF_68) | Outcome measures did not meet criteria |
| [Volandes, Paasche-Orlow, *et al.* (2009)](#_ENREF_69) | Outcome measures did not meet criteria |
